# Solitary Fibrous Tumor of the Umbilical Region in a Pediatric Patient

**DOI:** 10.31486/toj.22.0004

**Published:** 2022

**Authors:** Shruti Vaswani, Sudeep Khera, Arvind Sinha

**Affiliations:** ^1^ Department of Pathology and Laboratory Medicine, All India Institute of Medical Sciences, Jodhpur, Rajasthan, India; ^2^ Department of Paediatric Surgery, All India Institute of Medical Sciences, Jodhpur, Rajasthan, India

**Keywords:** *Molecular diagnostic techniques*, *solitary fibrous tumors*, *umbilicus*

## Abstract

**Background:** Fibrous tumors are rare tumors of mesenchymal origin arising in the serosal surfaces within the body. Although commonly seen in adults, solitary fibrous tumors rarely occur in children. Histopathology and immunohistochemistry are the methods of choice for diagnosing solitary fibrous tumors.

**Case Report:** A 2-year-old male presented with a swelling over the umbilicus for the prior 8 months. The umbilical mass was excised and sent for histopathologic examination. The skin-covered greyish soft tissue mass measured 6 × 5.5 × 4.5 cm, and the cut surface showed a homogenous greyish growth. On microscopic examination, a predominantly well-circumscribed encapsulated tumor was noted, with spindle shaped cells arranged in a haphazard manner and ectatic vascular channels. The cells were immunoreactive for CD34 and signal transducer and activator of transcription 6 (STAT6) and negative for smooth muscle actin, desmin, myogenin, MyoD1, CD99, epithelial membrane antigen, and beta-catenin.

**Conclusion:** The aim of this case is to make clinicians aware of the umbilicus as a rare site of solitary fibrous tumor in children and the diagnostic importance of STAT6.

## INTRODUCTION

Fibrous tumors are rare tumors of mesenchymal origin that arise in the serosa,^1^ visceral organs, and soft tissues.^2^ In adults, most fibrous tumors are benign.^3,4^ The incidence of solitary fibrous tumors in children is rare.^5^ Solitary fibrous tumors are well-circumscribed, slow-growing lesions for which the treatment of choice is surgical resection. Approximately 15% to 20% of cases have been reported to recur or metastasize.^6^ Parameters that contribute to recurrence and/or metastasis are tumor size, cellularity, nuclear pleomorphism, hemorrhage, and necrosis.^3^ We present a case of fibrous tumor in a pediatric patient presenting with a bulge in the umbilical region, a rare site in this age group.

## CASE REPORT

A 2-year-old male presented with swelling over the umbilicus for the prior 8 months. The swelling was initially small but gradually increased in size. The mass did not change size with changing positions, coughing, or straining and was not associated with any discharge. The patient had no vomiting, fever, abdominal distension, difficulty in micturition, diarrhea, constipation, or swelling anywhere else in the body. Because umbilical hernia is the most common umbilical disorder in this age group,^7^ a clinical diagnosis of umbilical hernia was considered.

Laboratory workup revealed hemoglobin of 10.9 g/dL (reference, 12.5 ± 1.5 g/dL), total leukocyte count of 11.78 K/mm^3^ (reference, 10 ± 5 K/mm^3^), and platelet count of 390 K/mm^3^ (reference range, 200-490 K/mm^3^).^8^ No radiologic imaging was done at our center. Imaging was done at an outside imaging center, but the parents of the patient lost the report, so we did not have access to the imaging and differentials offered with the imaging studies.

**Figure 1.**
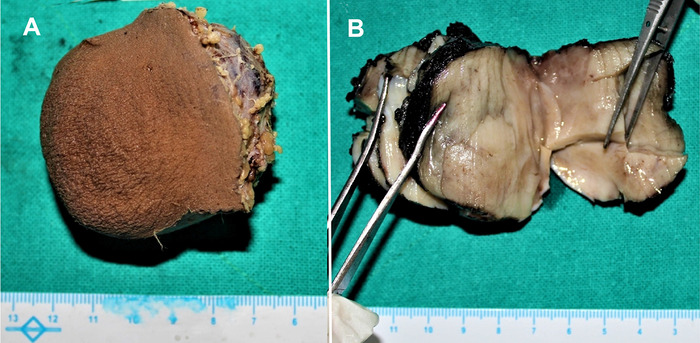
Gross examination revealed (A) a skin-covered soft tissue mass and (B) homogenous greyish white glistening areas on the cut section.

**Figure 2.**
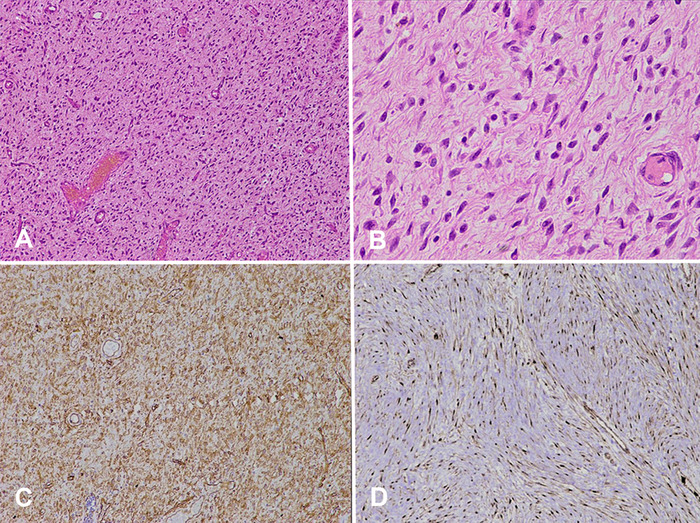
Solitary fibrous tumor shows a proliferation of spindle cells without a characteristic pattern and **hemangiopericytoma** vascular pattern with (A) hematoxylin and eosin (H&E) stain, magnification ×10, (B) H&E stain, magnification ×40, (C) diffuse membranous immunohistochemical staining of CD34 (magnification ×10), and (D) strong diffuse nuclear immunohistochemical staining of STAT6 (magnification ×10).

Intraoperatively, the surgeon did not find a hernial sac. The umbilical mass was excised and sent for histopathologic examination. The skin-covered greyish soft tissue mass measured 6 × 5.5 × 4.5 cm. The cut surface showed a homogenous greyish encapsulated growth that reached up to the deep resection margin and the skin margin. No heterogenous areas were identified (Figure 1). On microscopic examination, a predominantly well-circumscribed encapsulated tumor was noted, with spindle-shaped cells arranged in a haphazard manner and ectatic vascular channels (Figures 2A and 2B). The cells exhibited mildly hyperchromatic nuclei with indistinct cell borders, punctate nucleoli, and a moderate amount of pale eosinophilic cytoplasm. Immature fibroblastic proliferation was seen condensing around the perivascular region. A sprinkling of inflammatory cells composed of plasma cells, mast cells, and lymphocytes was also noted. Mitosis was <1/10 high-power fields (reference, >4/10 high-power fields in malignant solitary fibrous tumors^3^). No necrosis was seen.

Immunohistochemically, the tumor cells showed strong diffuse positivity for CD34 (Figure 2C) and signal transducer and activator of transcription 6 (STAT6) (Figure 2D). The tumor cells were negative for smooth muscle actin (SMA), desmin, myogenin, MyoD1, CD99, epithelial membrane antigen (EMA), and beta-catenin. The Ki-67 proliferation index was 2% to 3% only. Based on morphologic and immunohistochemical reactions, the mass was reported as a solitary fibrous tumor. At 1 year postoperatively, the patient remained disease-free.

## DISCUSSION

Solitary fibrous tumors are tumors of fibroblastic origin with spindle cells arranged without a characteristic pattern within a collagenous stroma along with a framework of staghorn-like vessels (hemangiopericytoma pattern).^9^ The word hemangiopericytoma was coined in 1942 by Stout and Murray for tumors located in the buttock, retroperitoneum, and thigh.^10^ They postulated that these tumors originated from pericytic-modified smooth muscle cells situated around blood vessels. Hemangiopericytomas and solitary fibrous tumors were merged in the fourth edition of the World Health Organization classification of tumors of soft tissue,^11^ and since then, the term hemangiopericytoma has been interchangeably used with the term solitary fibrous tumor.^12^ These tumors have been reported to occur commonly in the pleura, but other common sites include the mediastinum, peritoneum, extremities, and orbit.^13^ The incidence is particularly rare in pediatric patients according to the literature.^5^ These tumors are mostly benign. The few malignant cases that have been reported have shown metastasis to the bones, lungs, and liver.^13^ The literature suggests that reports of recurrence and metastasis occur in tumors >10 cm in diameter.^14^ Kanamori et al suggest that complete surgical resection is the most effective treatment modality for these tumors.^5^

Solitary fibrous tumors have been reported to show immunoreactivity for CD34, Bcl-2, and CD99.^9^ The discovery of gene fusion between NAB2 and STAT6 that leads to the upregulation of STAT6, a novel immunohistochemical marker, has been shown to be of diagnostic importance in these tumors.^6^

Tan et al examined 30 years of archives from 3 pediatric hospitals and identified 18 solitary fibrous tumor diagnoses.^9^ Of the 18 tumors, only 3 tumors from 2 patients demonstrated positive STAT6 staining and the typical histology and immunophenotype of solitary fibrous tumors. The remaining 15 tumors were reclassified as other types of soft tissue tumors after application of STAT6 immunohistochemistry, highlighting the rarity of solitary fibrous tumors in children and the importance of STAT6 as the diagnostic marker. None of the patients in the Tan et al study presented with the umbilicus as the site of tumor.^9^

Umbilical masses in children commonly include umbilical hernias, umbilical granulomas, remnants of the omphalo-mesenteric duct and urachus, and benign soft tissue masses, such as epidermoid cysts.^15^

Umbilical hernia commonly presents during the first few months of life after umbilical cord separation. A sac lined by peritoneum protrudes through an opening in the deep fascia of the abdomen. The protrusion becomes prominent when the baby cries or strains and is usually painless. Most umbilical hernias spontaneously resolve by the age of 3 years.^7^ In the present case scenario, the umbilical mass did not show any movement on coughing or straining, which is unusual, so the clinician decided to excise the mass to avoid any unforeseen complications.

An umbilical granuloma is a nodule of tissue approximately 1 cm in size that might become apparent following umbilical cord separation and is usually pedunculated. The treatment of choice is application of topical silver nitrate.^7^

The omphalomesenteric duct is an in utero connection between the terminal ileum and umbilicus. In most cases, the duct disappears after birth, but a part of it may persist. The Meckel diverticulum, an out-pouching of ileum, is the most common omphalomesenteric duct remnant. The common clinical presentation in children is obstruction. The presence of ectopic tissue increases the chance of the diverticulum becoming symptomatic. Diverticulectomy may be the treatment of choice in these cases.^16^

The urachus connects the dome of the urinary bladder to the abdominal wall anteriorly at the level of the umbilicus.^17^ In utero, the urachus is a patent tube that closes after birth and forms the median umbilical ligament. Failure of the lumen to obliterate may result in conditions similar to omphalomesenteric remnants. A urachal cyst is a residual cyst without any communication to the bladder or umbilicus and is noted in the midline of the abdominal wall inferior to the umbilicus. It usually presents as a tender, swollen mass secondary to infection and should be excised.^7^

Epidermal cysts are benign skin lesions usually involving the scalp, face, neck, and back^18^ but might be seen at the umbilicus.^19^ They may rupture and extrude keratin into the dermis, inciting a foreign body reaction.^20^ These cysts are usually asymptomatic unless infected and may be excised to avoid infection.^21^

In the present case, efforts were made to rule out all other tumors of mesenchymal origin. Solitary fibrous tumors need to be histologically differentiated from similar spindle cell tumors such as malignant peripheral nerve sheath tumor, fibrosarcoma, leiomyoma/leiomyosarcoma, and malignant fibrous histiocytoma although these tumors are more common in adults than in children. In pediatric patients, solitary fibrous tumors should be differentiated from inflammatory myofibroblastic tumors.^5^

Benign solitary fibrous tumors also need to be distinguished from malignant solitary fibrous tumors. Morphologic signs of malignancy include high mitoses (>4/10 high-power fields), moderate to marked nuclear pleomorphism, areas of high cellularity with nuclear crowding and overlapping, and the presence of necrosis or hemorrhage along with areas of stromal and/or vascular invasion.^3^ Our patient's tumor had none of these features.

Various immunohistochemical markers were performed to rule out other spindle cell tumors. SMA and desmin ruled out smooth muscle tumors. MyoD1 and myogenin ruled out skeletal muscle tumors. CD99 and EMA ruled out synovial sarcoma, and beta-catenin ruled out desmoid fibromatosis.^22^

To avoid overdiagnosis or underdiagnosis, appropriate morphologic and immunohistochemical features, as well as clinical and radiologic details, should be kept in mind while making the diagnosis.

As the tumor in our patient was superficial in location, we suggest that the origin of the tumor was the anterior abdominal wall or the umbilical cord remnant; however, because no radiologic investigation was done at our center, the exact site of origin could not be determined.

## CONCLUSION

Solitary fibrous tumors in pediatric patients are rare, and the umbilicus is a rare site of origin. These tumors commonly originate from pleural surfaces but can occur at any site in the body. Histopathology and immunohistochemistry are the methods of choice for diagnosing solitary fibrous tumors. These tumors show haphazardly arranged spindle cells and vascular channels with hemangiopericytoma-like branching patterns showing immunoreactivity for STAT6 and CD34 markers. Surgical excision is currently the recommended therapy in the pediatric population.

## Notes

*This article meets the Accreditation Council for Graduate Medical Education and the American Board of Medical Specialties Maintenance of Certification competencies for Patient Care and Medical Knowledge.*
